# Alcohol-induced deficits in reactive control of response selection and inhibition are counteracted by a seemingly paradox increase in proactive control

**DOI:** 10.1038/s41598-023-28012-5

**Published:** 2023-01-19

**Authors:** Ann-Kathrin Stock, Paul Wendiggensen, Filippo Ghin, Christian Beste

**Affiliations:** 1 Cognitive Neurophysiology, Department of Child and Adolescent Psychiatry, Faculty of Medicine of the TU Dresden, Schubertstrasse 42, 01309 Dresden, Germany; 2 University Neuropsychology Center, Faculty of Medicine, TU Dresden, Dresden, Germany; 3 Faculty of Psychology, TU Dresden, Dresden, Germany

**Keywords:** Neuroscience, Cognitive neuroscience, Human behaviour

## Abstract

High-dose alcohol intoxication reduces cognitive control, including inhibition. Although inhibition deficits may contribute to the behavioral deficits commonly observed in alcohol use disorder (AUD), many questions about potentially modulating factors have remained unanswered. We examined the effects of experimentally induced high-dose alcohol intoxication (~ 1.1 ‰) on the interplay between controlled vs. automatic response selection and inhibition in healthy young men. A holistic EEG-based theta activity analysis that considered both reactive control during task performance and preceding proactive control processes was run. It revealed a previously unknown seesaw relationship, with decreased reactive control, but paradoxically increased proactive control. Most importantly, alcohol-induced increases in proactive occipital theta band power were associated with reductions in negative alcohol effects on reactive control processes associated with decreased activity in the SMA and medial frontal cortex. Our findings demonstrate that research should not solely focus on immediate effects during task performance. Aside from differential neurobiochemical and neuroanatomical effects of alcohol, it is also conceivable that proactive control may have been recruited in a (secondary) response to compensate for alcohol-induced impairments in reactive control. Against this background, it could be promising to investigate changes in such compensatory mechanisms in pronounced alcohol-associated inhibition deficits, like in AUD patients.

## Introduction

Alcohol use disorder (AUD) is a prevalent problem in many (western) societies, and much research effort has been invested to identify neural mechanisms underlying both the loss and regaining of control over drug intake^1^. In order to better support AUD patients in regaining control, it is crucial to better understand the factors modulating cognitive control, especially inhibition^2^, and determine the underlying neural mechanisms and functional neuroanatomical correlates. It is however also crucial to understand how mechanisms of inhibitory control are modulated by acute high-dose alcohol intoxication^3^, as frequent binge drinking increases the likelihood of later developing AUD^4,5^. Several studies examined how inhibitory control processes and their underlying neurophysiological mechanisms are modulated by high-dose acute alcohol intoxication of ~ 1.2‰^3,6–8^. In this context, one recent study focused on the interplay of automatic and controlled processes during response inhibition^3^. This was done because the relative strength of automatic and controlled response tendencies modulates inhibitory control processes^9,10^ known to be altered in AUD^11^ and may further be essential to better understand basic drug consumption in AUD^12^. This previous study demonstrated that the interplay between controlled and automatic processes might be somewhat less impaired by acute alcohol intoxication than cognitive control alone, thus potentially helping to maintain inhibitory functions during intoxication^3^. Even though this study also examined some neurophysiological (EEG) correlates of this phenomenon, certain key functional aspects had remained unclear: specifically, it had remained unclear which functional neuroanatomical structures are modulated by acute alcohol intoxication effects during the interplay between controlled and automatic processes in response inhibition. Yet, knowledge about the involved functional neuroanatomy is crucial to develop and optimize non-invasive brain stimulation approaches, which are increasingly considered for (additional) treatment of AUD^13,14^. In this context, it is also essential to consider oscillatory neural activity, as it is relevant to neural information processing^15–18^ and may be directly targeted by possibly relevant brain stimulation approaches for the treatment of addiction (e.g., transcranial alternating current stimulation)^19,20^. To address these critical and previously unanswered aspects, we re-analyzed EEG data from the study by Chmielewski et al.^3^. Doing so, an emphasis was placed on theta band activity and beamforming analyses to delineate the functional neuroanatomical structures reflecting acute alcohol intoxication effects during the interplay between controlled and automatic processes in response inhibition.

Several frequency bands have been associated with different facets of inhibitory control processes^21^. In this regard, gamma frequency seems to be associated with both response execution and response inhibition preparation if an exogenous stop-signal is presented (i.e., action cancellation)^22–24^, but there are no studies providing evidence for altered gamma oscillations in the context of alcohol intoxication and response inhibition. Furthermore, evidence suggests that beta frequency is important for motor action preparation and maintenance of the current motor action^21^, as well as for modulating the threshold for motor execution/inhibition^25^. Yet, the role of beta oscillations in alcohol intoxication-induced changes of the interplay between automaticity and control was previously found to only indicate changes in motor cortex activation, while cognitive conflict was reflected by the theta band^26^. Crucially, this is also in line with findings linking intoxication-induced alterations of the theta band to intoxication-induced response inhibition deficits^6^. Theta oscillations have been found to play a critical role in response inhibition processes^27^. Although theta band activity has been associated with a range of higher cognitive functions such as working memory, memory encoding, top-down control^27^ and social cognition^28^, it has been demonstrated that its activity reflects the need for control that has to be exerted for response inhibition, and is thus critical for flexible goal-directed behavior. Furthermore, theta band activity has been shown to be a key neurophysiological index of the modulation of the level of automaticity in inhibition control^21,27,29–31^ and to reflect relevant effects in the task investigated in the current study^32^. Against this background, dedicated analyses are conducted for this frequency band to investigate the interplay of automatic and controlled processes in response inhibition during alcohol intoxication.

The experimental paradigm employed by Chmielewski et al.^3^ combined a Simon task measuring response selection and conflict monitoring^33^ with a Go/Nogo task measuring inhibitory control. The Simon-type variation of spatial stimulus and response locations allows to compare rather automatic stimulus–response selection processes (in spatially congruent trials) and more controlled stimulus–response selection processes (in spatially incongruent trials)^34^. The additional inclusion of Nogo trials in this experimental setup allows to assess differences in response inhibition in more automatic vs. more controlled contexts. Considering the role of medial and superior frontal theta band activity during conflict monitoring^27,31,35^ and inhibitory control^21,36^, as well as the functional link between declined theta band activity and inhibitory control during acute alcohol intoxication^3,6–8^, we hypothesized that alcohol intoxication effects decrease theta band activity in superior frontal and medial frontal cortices during response execution and inhibition trials.

However, it is essential to not only focus on those reactive forms of control, but also consider proactive forms of cognitive control in this context. Proactive control is exerted to prepare the cognitive system for an upcoming task and cognitive demands^37^. Recent data suggest that such proactive control processes are not only exerted when explicitly triggered (e.g., by information from the environment). Instead, individuals build expectancies about upcoming events and the likelihood to engage, e.g., in inhibitory control. This establishes a proactive control state in between the inhibitory requirements/trials, which also reflects different task demands^29,38^. Intriguingly, such proactive control states are related to theta band activity^39^ and, even more importantly, are predictive for the neurophysiological processes that occur while (subsequently) engaging in inhibitory control^29,38^. This shows that understanding how acute alcohol intoxication affects proactive control preceding the actual response inhibition is probably as important as understanding the directly inhibition-associated processes. Reactive and proactive inhibition have been successfully investigated with several behavioral experimental desings^40–42^. For example, it has been shown that a reaching version of the stop-signal task allows to investigate how different cognitive contexts (i.e., presence of infrequent stop signals versus when no stop-signal is presented), where reactive inhibition can be calculated by measuring the extent of stop-signal reaction time at different stop-signal delays, while proactive inhibition can be calculated by comparing reaction times and movement times during no-stop trials in the stop-signal condition versus when only go-trials occur^40^. This behavioral approach has been proven to sensibly measure reactive and proactive inhibition processes in both healthy^40^ and clinical populations^41–43^. Yet, to better understand and differentiate the neural processes underlying inhibitory control requires an in-depth and holistic analysis of the inter-dependencies between theta band activity during both active inhibition and pro-active control processes. This is all the more relevant when considering the need to better understand the role of proactive control for alcohol consumption and abuse. Given that the currently available data on alcohol and proactive control is very heterogeneous^44–48^, no clear-cut hypotheses can be informed on how alcohol intoxication modulates proactive control-related theta band activity in the pre-trial period (i.e., before the individual has to probably exert inhibitory control). Hence, we ran exploratory data analyses on theta band activity to learn which functional neuroanatomical structures are associated with alcohol effects on pre-trial pro-active control. Since proactive control implies some form of top-down attentional control^37^ and scanning the environment for upcoming effects, several brain areas may be functionally relevant: There are sensory (occipital) regions involved in attentional scanning processes for upcoming motor control^49,50^. Inferior frontal regions likely serve overarching attentional functions for upcoming events^51,52^. Ventral prefrontal areas may also be relevant as they have previously been associated with proactive control occurring before the possible engagement in response inhibition^29^. Regardless of the specific brain regions associated with theta band-related alcohol intoxication effects in the pre-trial interval, we hypothesized that theta band activity in the pre-trial interval predicts (i.e., correlates with) the expected alcohol intoxication-induced decrease of theta band activity in superior frontal and medial frontal cortices during response execution and inhibition trials.

## Results

### Experimentally induced intoxication

The included N = 35 participants were on average 180.3 ± 1.3 cm tall, weighed 73.8 ± 1.5 kg, and received 275 ± 4 mL of vodka (40 vol%). After the 30 min waiting period (after the end of consumption), participants had an average breath alcohol concentration (BrAC) of 1.10 ± 0.04‰. After completing the experiment, participants had a mean BrAC of 1.07 ± 0.03‰. The AUDIT scores did not correlate with any of the behavioral measures (all *p* > 0.05; Bonferroni-corrected). Additionally, there were no differences between the *N* = 27 individuals with AUDIT scores in zone I (0–8 points) and the *N* = 8 individuals who had AUDIT scores in zone II (9–15 points) on any behavioral measure (Mann–Whitney *U* tests; all *p* ≥ 0.146). Hence, the AUDIT scores were not used in the statistical analysis to correct for drinking habits.

### Behavioral data

**Figure 1:**
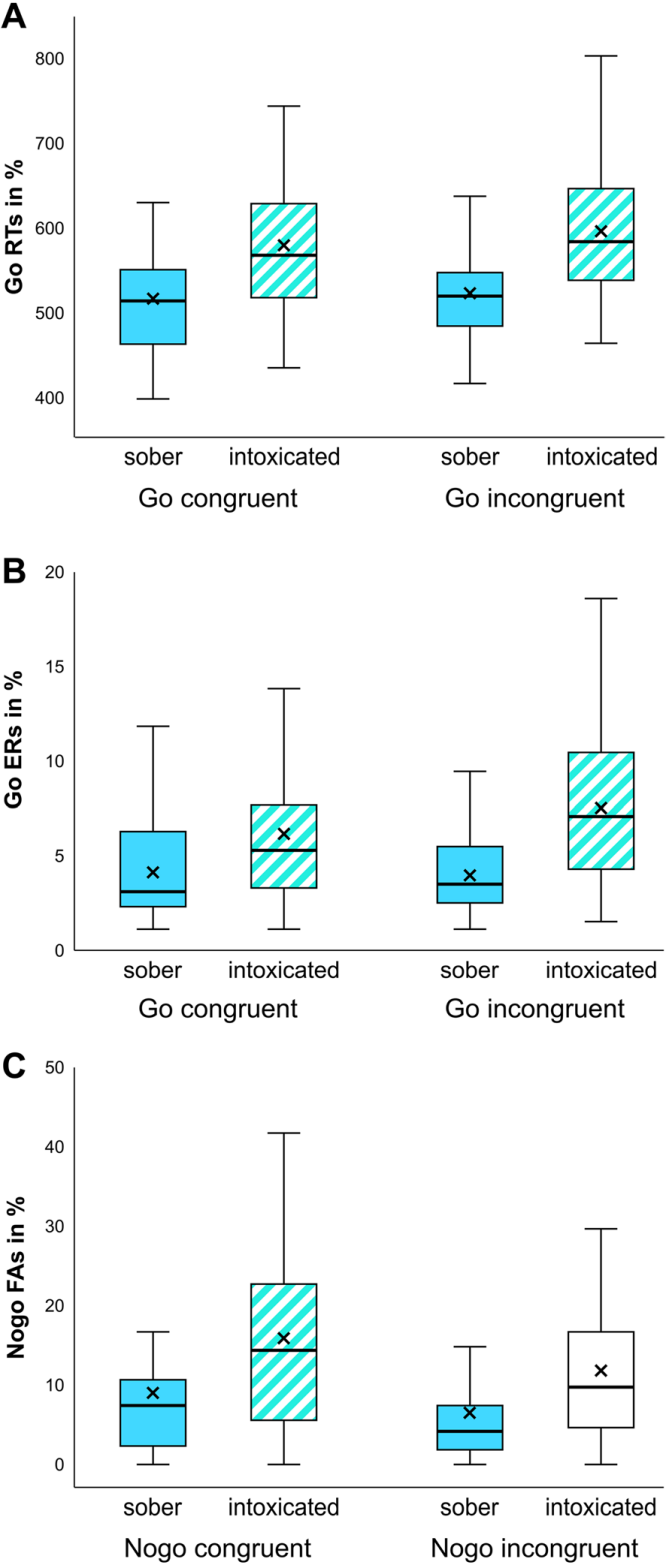
Illustration of the obtained behavioral data. (**A**) The top graph shows Go hit RTs in ms. Of note, the intoxication effect was significantly larger in incongruent than in congruent trials. (**B**) The middle graph shows commission and omission errors/Go error rates in percent. Of note, the intoxication effect was significantly larger in incongruent than in congruent trials. (**C**) The bottom graph shows comission errors/Nogo false alarm rates in percent. Participants performed significantly worse while intoxicated. Effects of appointment order are not illustrated.

Behavioral results for omission and commission errors in Go trials (summed up as “error rate”/ER), reaction times (RTs) for correct responses in Go trials, and commission errors (termed “false alarm”/FA rate, as in our previous publication) are shown in Fig. 1.

In the repeated measures ANOVA for Go trial RTs, “congruency” (congruent vs. incongruent) and “intoxication status” (sober vs. intoxicated) were used as within-subject factors and “appointment order” (sober first vs. intoxicated first) was used as between-subject factor. Doing so, a main effect of congruency was found (F_(1,33)_ = 20.40; *p* < 0.001; *η*^2^_*p*_= 0.382), with slower responses in incongruent trials (545 ± 9 ms) compared to congruent trials (532 ± 10 ms). Additionally, a main effect of intoxication status was found (F_(1,33)_ = 47.91; *p* < 0.001; *η*^2^_*p*_ = 0.592), where intoxication was associated with slower responses (567 ± 12 ms) compared to the sober appointment (510 ± 9 ms). There was a main effect of appointment order (F_(1,33)_ = 8.66, *p* = 0.006; *η*^2^_*p*_ = 0.208; first sober: 510 ± 14 ms, first intoxicated: 567 ± 13 ms) and an interaction of intoxication status and appointment order (F_(1,33)_ = 13.67; *p* < 0.001; *η*^2^_*p*_ = 0.293). Post-hoc t tests showed that participants who were intoxicated during the first appointment responded slower compared to participants who were sober on the first appointment, but only during intoxication (*t*_(33)_ = 3.73; *p* < 0.001; intoxicated first: 610 ± 17 ms; intoxicated second: 523 ± 16 ms) and not while sober (*t*_(33)_ = 1.45; *p* = 0.156; sober first = 497 ± 12 ms; sober second: 523 ± 14 ms). Furthermore, there was an interaction between intoxication status and congruency (F_(1,33)_ = 6.56; *p* = 0.015; *η*^2^_*p*_ = 0.166). Post-hoc paired t tests revealed that the intoxication effect (intoxicated minus sober) was significantly larger in incongruent Go trials (63 ± 10 ms) than in congruent Go trials (56 ± 10 ms) (*t*_(34)_ = 2.65; *p* = 0.012). All other effects were non-significant (all F ≤ 2.15; *p* ≥ 0.152).

For the ER in Go trials, the repeated measures ANOVA again used “congruency” and “intoxication status” as within-subject factors and “appointment order” as between-subject factor. Doing so revealed a significant main effect of congruency (F_(1,33)_ = 4.35; *p* = 0.045; *η*^2^_*p*_ = 0.116), where ER were higher in incongruent (4.5 ± 0.5%) than in congruent trials (3.6 ± 0.4%). A main effect of intoxication status was found (F_(1,33)_ = 10.52; *p* = 0.003; *η*^2^_*p*_ = 0.242), where ER were higher in the intoxicated (5.0 ± 0.6%) as compared to the sober appointment (3.0 ± 0.4%). Furthermore, an interaction effect of congruency and intoxication status was found (F_(1,33)_ = 4.17; *p* = 0.049, *η*^2^_*p*_ = 0.112). Post-hoc t-test revealed that the intoxication effect (intoxicated minus sober) borderlined on a larger effect in the incongruent (2.5% ± 0.7) as compared to the congruent condition (1.4% ± 0.6) (*t*_(34)_ = 2.02; *p* = 0.051). All other effects were non-significant (all F ≤ 0.37; *p* ≥ 0.546).

On the Nogo trials, a repeated measures ANOVA on the false FA rate was run with “congruency” and “intoxication status” as within-subject factors and “appointment order” as between-subject factor. This showed a main effect of congruency (F_(1,33)_ = 30.06; *p* < 0.001, *η*^2^_*p*_ = 0.477), where performance was worse (i.e., higher FA rate) in congruent (11.9 ± 1.3%) as compared to incongruent trials (8.5 ± 1.0%). Moreover, a main effect of intoxication status was found (F_(1,33)_ = 17.96, *p* < 0.001; *η*^2^_*p*_ = 0.352), as participants performed better (i.e., lower FA rates) during the sober (6.9 ± 1.0%) than during the intoxicated (13.4 ± 1.6%) appointment. All other effects were non-significant (all F ≤ 2.50; *p* ≥ 0.123).

### Neurophysiology

**Figure 2:**
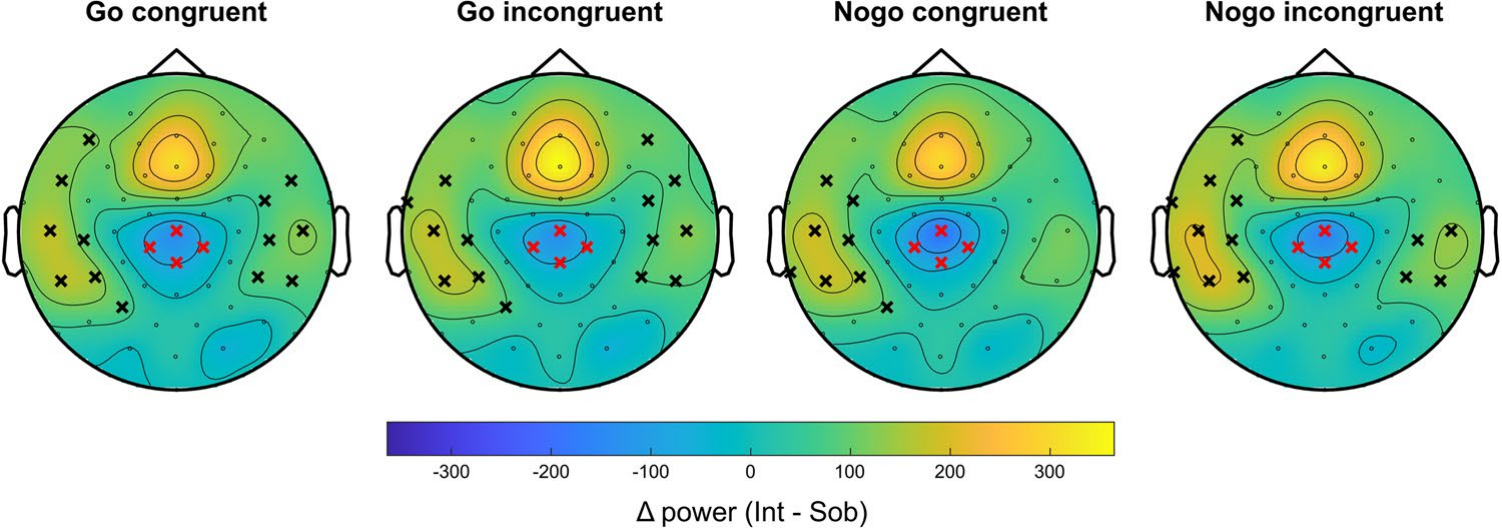
Depiction of the intoxication effect (intoxicated minus sober) on theta band activity at the electrode level. In all four experimental conditions, the intoxicated participants showed significantly higher theta power at lateral electrodes (marked with a black “x”) and significantly lower theta power at parieto-central electrodes (marked with a red “x”).

In the pre-trial interval (− 600 to 0 ms relative to stimulus onset), a cluster-based permutation test with 1000 Monte Carlo randomizations was run to compare the sensor/electrode-level theta power (as a measure of theta band activity) of the intoxicated and sober conditions. The power difference was statistically significant (*p* = 0.002), revealing a higher theta activity in the intoxicated than the sober state spanning all electrode locations. In the within-trial interval (150 to 750 ms relative to stimulus onset), cluster-based permutation tests were used to separately compare the theta power (as a measure of theta band activity) between the intoxicated and sober conditions for all four trial types. In the Go congruent condition, statistically higher theta band activity in the intoxicated than the sober condition (*p* = 0.013) was found at lateral electrodes. However, lower theta band activity in the intoxicated condition, as compared to the sober condition, was found (*p* = 0.010) at parieto-central electrodes 'Cz,' 'CP1',' CP2', 'CPz'. For the Go incongruent condition, significantly higher (*p* = 0.011, lateral electrodes) and lower (*p* = 0.024, parieto-central electrodes) theta band activity was identified in the intoxicated condition. Similar patterns of theta band activity modulations emerged in congruent and incongruent Nogo trials (all *p* < 0.021), although in contrast to the Go condition, the effect seems to be lateralized to the left hemisphere. The significant electrodes are visualized on the power difference maps (intoxicated minus sober) in Fig. 2.

**Figure 3:**
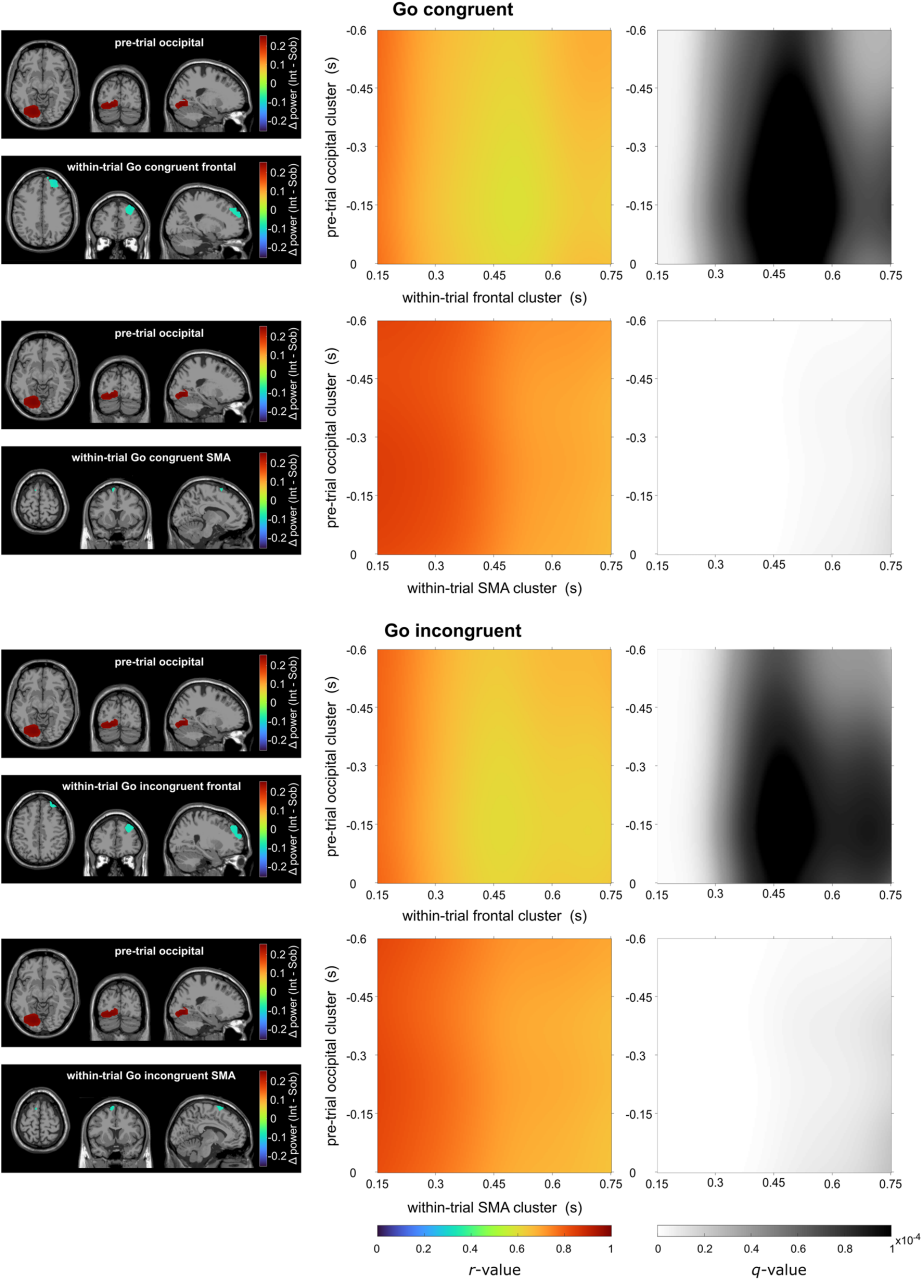
Illustration of the intoxication effect in Go trials on the neuroanatomical source level. The left column depicts the brain areas underlying significant changes in theta band activity in the pretrial time window (occipital; intoxicated > sober) and within-trial time window (frontal and SMA, intoxicated < sober). The middle column depicts the correlation coefficient for the correlation between the theta band intoxication effects in the pre-trial (y-axis) and within-trial (x-axis) clusters which are depicted on the left. The right column depicts the associated significance value *q* (the FDR-corrected *p* value of each correlation), with brighter colors indicating a higher significance.

**Figure 4:**
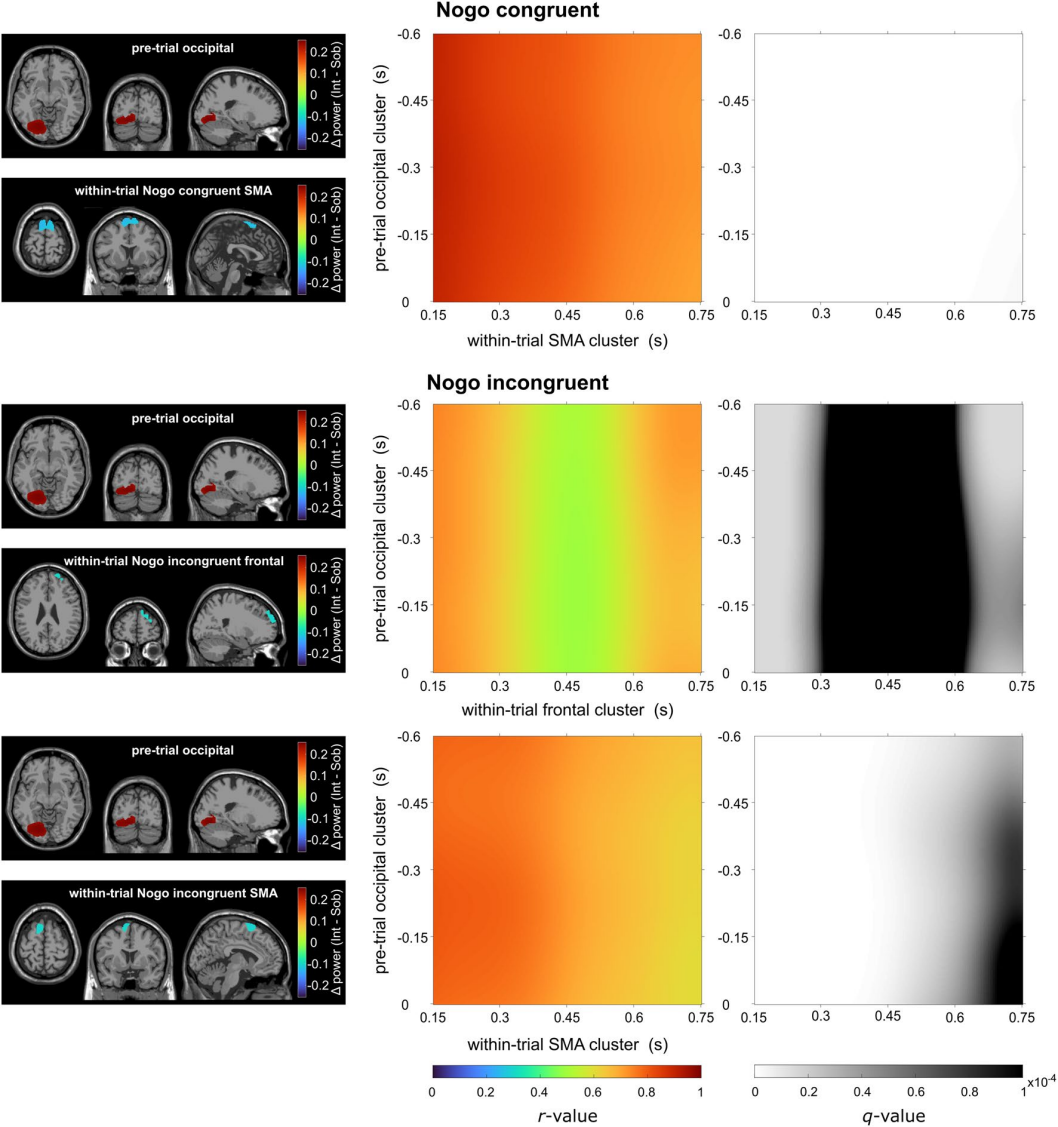
Illustration of the intoxication effect in Nogo trials on the neuroanatomical source level. The left column depicts the brain areas underlying significant changes in theta band activity in the pretrial time window (occipital; intoxicated > sober) and within-trial time window (frontal for incongruent Nogo only, and SMA for both Nogo conditions, intoxicated < sober). The middle column depicts the correlation coefficient for the correlation between the theta band intoxication effects in the pre-trial (y-axis) and within-trial (x-axis) clusters depicted on the left. The right column depicts the associated significance value *q* (the FDR-corrected *p* value of each correlation), with brighter colors indicating a higher significance.

On the source-reconstructed theta band activity level, the DBSCAN algorithm identified a cluster in occipital areas in the pre-trial interval, which shows more theta band activity in the intoxicated state than in the sober state. For the within-trial period, clusters in the medial-superior cortex and the supplementary motor area (SMA) were identified for the congruent and incongruent Go, as well as for the incongruent Nogo condition. For the within-trial congruent Nogo condition, only a cluster in the SMA was found. All within-trial clusters showed smaller theta band activity in the intoxicated state than in the sober state. The clusters are visualized left of the correlations in Figs. 3 and 4 for Go and Nogo conditions, respectively.

The correlations between the intoxication effect in the pre-trial and the intoxication effect in the within-trial period are also visualized in Figs. 3 and 4. For each correlation matrix, the false discovery rate (FDR) of the *p* values was adjusted using the Benjamini-Hochberg method, resulting in *q *values (for more details, see methods). For the Go congruent condition (compare Fig. 3), all time points of the pre-trial activity in the occipital cluster correlated positively with the within-trial superior frontal cluster, especially in the time frames from 150 to 350 ms and after 650 ms (relative to stimulus onset, *r*_*max*_ = 0.78, *r*_*min*_ = 0.62, within area *q* < 0.0001). The correlation is less significant in the period centered around the 500 ms mark (i.e., shortly before the button press). The correlation between the pre-trial occipital cluster and the within-trial SMA cluster is highly significant as well. The correlation plot shows a pattern where all time points of the intoxication effect in the pre-trial and the within-trial intervals correlate positively, with a reduced magnitude over time (*r*_*max*_ = 0.85, *r*_*min*_ = 0.66; within *q* < 0.0001). For the Go incongruent condition (compare Fig. 3), all time points of the pre-trial occipital cluster correlated positively with the frontal within-trial cluster in the time frames from 150 to approximately 350 ms (relative to stimulus onset, *r*_*max*_ = 0.79, *r*_*min*_ = 0.61; within area *q* < 0.0001). Correlation magnitudes are lower around the 500 ms (i.e., shortly before the response). Additionally, the intoxication effect in the pre-trial occipital and the within-trial SMA cluster correlated, with higher correlations at the start of the within-trial period (150 ms relative to stimulus presentation) and lower correlations at the end (*r*_*max*_ = 0.83, *r*_*min*_ = 0.65; within *q* < 0.0001).

In the Nogo congruent condition (compare Fig. 4), all time points of the within-trial intoxication effect in the SMA cluster correlated positively with the occipital pre-trial intoxication effect. However, correlations were higher at the start of the within-trial period (*r*_*max*_ = 0.91) and lower towards the end (*r*_*min*_ = 0.70). Concerning the Nogo incongruent trials, the intoxication effect in the pre-trial occipital cluster correlated with the intoxication effect in the frontal cluster in the within-trial time frames from 150 to 300 ms and 650 to 750 ms (relative to stimulus onset, *r*_*max*_ = 0.74, *r*_*min*_ = 0.63; within *q* < 0.0001). The intoxication effect of the within-trial SMA cluster and the pre-trial occipital cluster correlated at all time points, with higher correlations at the beginning of the within-trial period (150 ms) and lower correlations towards the end of the trial (*r*_*max*_ = 0.81, *r*_*min*_ = 0.61; within *q* < 0.0001).

## Discussion

In the current study, we examined the effects of an experimentally induced high-dose alcohol intoxication (binge drinking) on neurophysiological processes associated with the interplay between controlled and automatic response selection during response inhibition. In particular, we focused on the theta frequency band and underlying functional neuroanatomical structures. Instead of merely examining neurophysiological processes during response execution and inhibition, we performed a more holistic analysis that also considered the relevance of proactive control processes. For this, we investigated the inter-relationship between pre-trial and within-trial neural activity. Doing so significantly extend previous data analyses performed on same data set^3^. To account for this, we focused the discussion in the current study on the findings of the neurophysiological data analysis and kindly refer to the previous publication for a more extensive discussion of the behavioral data^3^.

Matching our first hypothesis, we found that during task performance (i.e., in the within-trial interval) alcohol intoxication decreased theta band activity at the most commonly investigated mid-central electrodes. Importantly, this was the case in all investigated situations, i.e., during response execution and response inhibition as well as for congruent and incongruent trial types. The beamforming analyses revealed that regions in the middle frontal gyrus (MFG), as well as the superior frontal gyrus (SFG) and the supplementary motor area (SMA) were associated with these alcohol intoxication effects. Theta band activity in superior frontal and medial frontal regions is essential for cognitive control processes during the execution of actions and the inhibition of responses^27,31,35,53^. The likely reason for this is that medial/superior frontal theta band activity is ideally suited to integrate sensory information with motor commands during response selection^15,27^. Thus, the current data thus suggests that theta band activity-related cognitive control processes are diminished in superior prefrontal structures as an effect of high-dose alcohol intoxication. The finding that the SMA revealed modulations in all investigated trials types (Go and Nogo, congruent and incongruent) suggests that this brain region may be a particularly important target region for attempts to modulate cognitive control processes in alcohol-related conditions via non-invasive brain stimulation approaches (in)directly targeting theta band activity. Since alcohol diminished theta-related cognitive control activity in the SMA, protocols aiming to actively increase mid-frontal theta band activity are likely most relevant for potential AUD treatment approaches. Most importantly, however, our data clearly show that alcohol intoxication effects on within-trial (cognitive control-related) theta band activity cannot be seen in isolation from alcohol intoxication effects on proactive control processes in the pre-trial period.

Opposed to the alcohol-induced theta band activity decrease in the within-trial, alcohol intoxication actually increased theta band activity in the pre-trial time period, as compared to the sober state. The beamforming analysis showed that theta band activity in the left lingual gyrus (occipital cortex) was stronger in the intoxicated state than in the sober state. Previous findings suggest that the functional significance of pre-trial theta band activity lies in some form of proactive control^29,38^ exerted to prepare the cognitive system for an upcoming task and associated cognitive demands^37^. Interestingly, preparatory attentional scanning processes for upcoming motor control have been attributed to occipital (lingual gyrus) regions^49,50^. The current data suggest that such proactive attentional scanning processes for upcoming demands are intensified by high-dose alcohol intoxication. Hence, the data point towards a dissociation of alcohol effects, with increased proactive preparatory (pre-trial interval) processing and decreased reactive response selection and inhibition processes (within-trial interval). Intriguingly, there are substantial positive correlations between alcohol intoxication effects in the pre-trial and the within-trial theta band activity. The obtained positive correlations between the two time periods (explaining between 36 and 64% of the variance) show that stronger alcohol-induced increases in pre-trial theta band activity (i.e., stronger preparatory processes as reflected by a positive intoxication effect) came along with less impairing (i.e., less negative) alcohol effects during response execution and inhibition—and vice versa. This relationship was evident in all examined experimental conditions and thus reflects a robust result independent of response selection and inhibition demands. However, there were slight fluctuations in the strength of correlations depending on the brain structure. Pre-trial occipital theta band activity appeared to be more strongly correlated with within-trial SMA activity, than with within-trial (pre)frontal activity. For the within-trial SMA theta band activity, the intoxication effect in the pre-trial occipital cluster correlates at all times, but the magnitude of the correlation slightly decreases with time. Since the SMA is known to be involved in both the generation of motor responses and in specifying response inhibition^54^, it could be speculated that the preparatory processes taking place during the pre-trial period support/foster the task-relevant response selection and inhibition processes driven by the SMA and thus have a protective effect on performance. In this context, the slight decline in this correlation towards the end of the within-trials could potentially be explained by a slight disengagement of SMA-associated control processes after task performance and the evaluation thereof have been finished^55^. Correlations between the pre-trial and (pre)frontal within-trial theta band activity differences were a little less pronounced and became smaller in the time interval around button press, or inhibition thereof (~ 500 ms). Importantly, this implies that the preparatory attentional scanning processes for upcoming motor control ascribed to occipital regions^49,50^ have a much smaller beneficial/protective effect on the cognitive control processes that are driven by (pre)frontal medial areas and required specifically around the time of response execution and inhibition. The medial frontal cortex has repeatedly been shown to play a key role for response selection and response inhibition^56^ as well as for performance monitoring and outcome evaluation^57.^ Reduced functionality of the medial (pre)frontal cortex has been associated with reduced goal-directed movements^58^, and reduced active inhibition of distracting information^59^. Given that the timing of the observed correlation decrease matched the mean response times, it would however be most plausible so assume that there are aspects of response selection and/or inhibition (rather than of result evaluation), which are driven by the medial (pre)frontal cortex and do not benefit from preparatory attentional processes in the same way as SMA-associated sub-processes of cognitive control.

Our findings point to a currently unknown seesaw relationship in alcohol intoxication effects between proactive control and reactive control. The theta band activity differences found during both response execution and response inhibition suggest that it is not sufficient to solely focus on immediate detrimental effects of alcohol intoxication in task performance, as often done in substance or addiction research. For example, the role of both proactive and reactive inhibition has received particular attention in clinical conditions characterized by poor inhibitory control functions^60^.

The main question raised by our findings is whether and how alcohol intoxication causes this paradox pattern of increased proactive and decreased reactive control. On the one hand, it could be possible that the complex neurobiochemical effects of alcohol have differential, and partly opposing effects on occipital and (pre)frontal brain regions, thus resulting in the facilitation of occipital processing at the cost of prefrontal processing. If this was true, it could be an example of the so-called "net zero-sum" principle of brain function^61,62^. It states that (cognitive) effects of neural activity modulations can have opposing indirect effects, as down-regulations (negative effects) in one part of the network can entail an up-regulation (positive effect) is other parts of the network and closely connected cognitive processes. The paradox increase in pre-trial occipital theta band activity may thus reflect an indirect effect of the prefrontal reactive control deficits based on the net-zero sum principle. The observed inverse relationship between proactive and reactive theta band processes may further support this. Given the observed inverse relationship of less pronounced reductions in frontal reactive control with increasing occipital proactive preparation and attentional gating processes, it could however also be possible that healthy individuals tend to recruit proactive control resources in order to compensate for alcohol-induced reductions in reactive cognitive control. If this was the case, it could be a promising new approach to extend the current focus in addiction research and investigate whether individuals with pronounced alcohol-associated inhibition deficits, like AUD patients, suffer from an (additional) decline in the recruitment of proactive control resources.

## Methods

Full details on the methods are given in the supplemental material.

### Participants

The current study sample was largely (but not completely) overlapping with a previous study^3^. It consisted of *N* = 35 (age 19–33; *M*_*age*_ = 24.89; *SD*_age_ = 3.08) young healthy participants with no history of neurological or psychiatric disease. Only males were included in the current study. Participants reported an AUDIT score of *M* = 6.89 (*SD* = 2.49). All participants provided written informed consent. The study was approved by the Ethics Commission of the Medical Faculty of the TU Dresden (EK 293082014) and conducted in accordance with the declaration of Helsinki.

### Experimental design and alcohol administration

**Figure 5:**
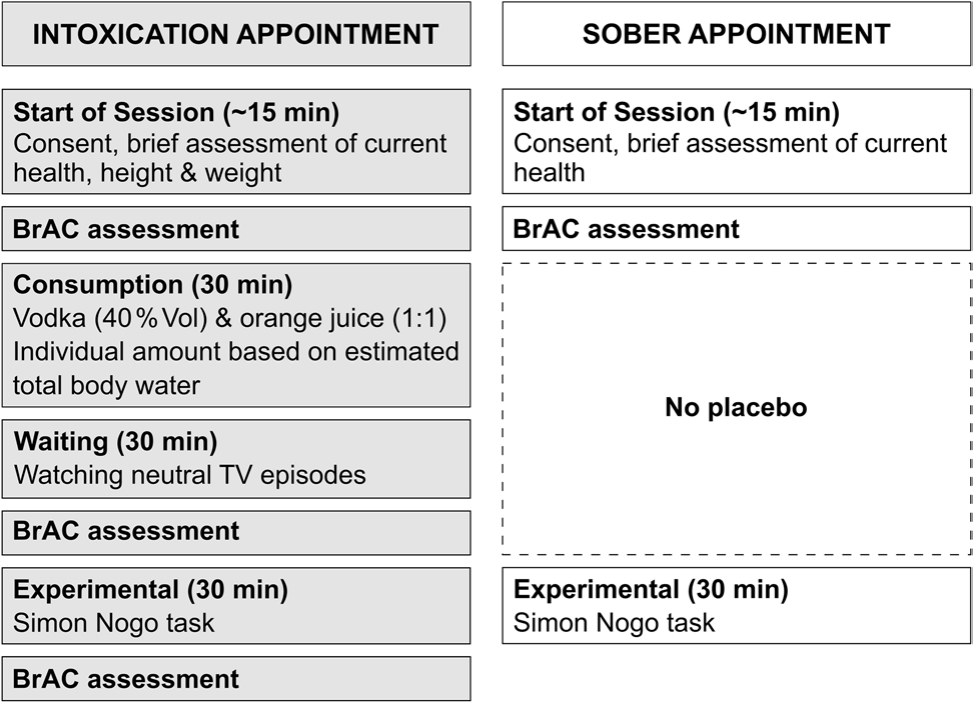
Illustration of the time course of both study appointments. The order of the appointments was randomized and there was a mandatory delay of no less than 2, and no more than 5 days between the appointments.

The study design (Fig. 5) and alcohol administration procedure were identical to previous studies (e.g., Stock et al.^63^). A within-subject design was used, where subjects were tested once sober and once intoxicated. The alcohol administration followed a fixed experimental procedure based on individual total body water estimates to reach a mean peak BrAC of 1.2 ‰ (for details, please refer to the supplement). BrAC levels were measured immediately before and after the task using the "Alcotest 3000" breathalyzer (Drägerwerk, Lübeck, Germany).

### Task

**Figure 6:**
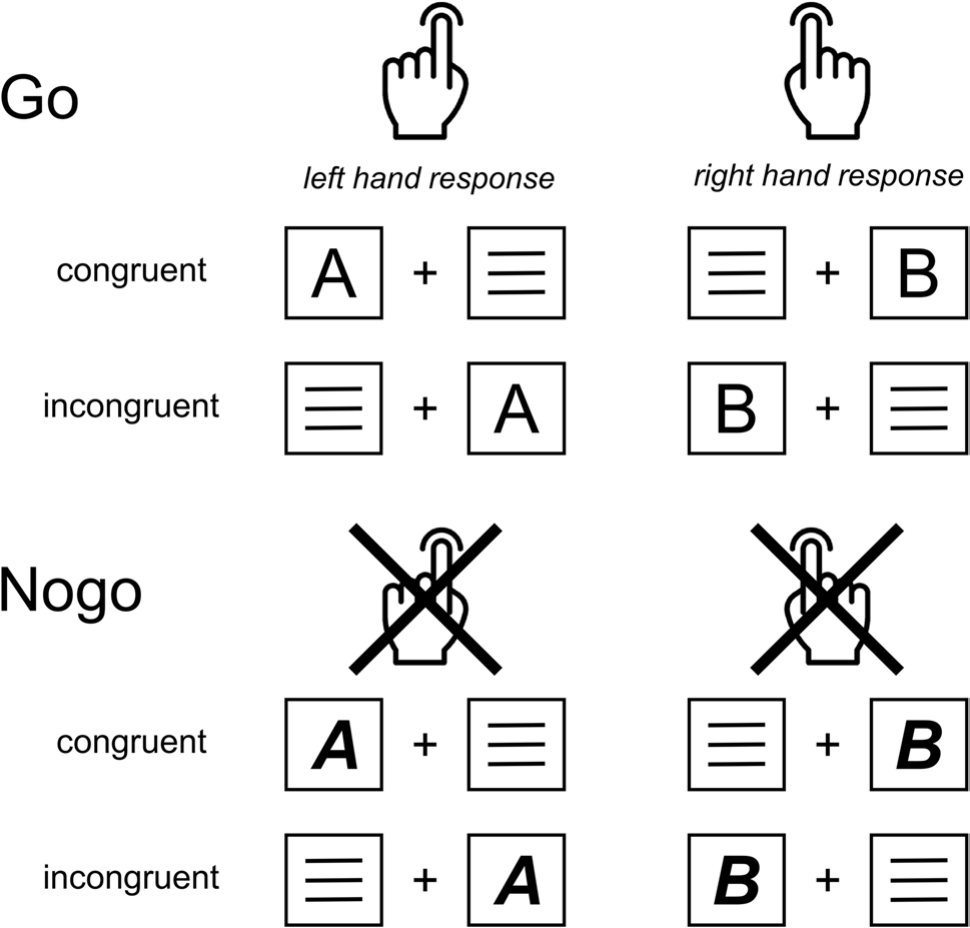
Illustration of the experimental paradigm. Go trials (see top panel) required a left hand response to the target stimulus “A” and a right hand response to the target stimulus “B”, irrespective of the side that they were presented on. Nogo trials (see bottom panel) were signaled by bold italic letters (“***A***” and “***B***”) and required the inhibition of all responses. Go trials were rated as “congruent” when target stimulus and responding hand were located on the same side, and rated as “incongruent” when they were located on contralateral sides. Nogo trials were labelled analogously to the Go trials, even though they did not require a motor response.

A combination of a Simon and Go/Nogo task^3,10^ was used*.* The paradigm is visualized in Fig. 6.

Each trial started with the 200 ms presentation of a lateralized target stimulus and a contralateral distractor and either ended with the first button press, or after 1200 ms, if no response was given. Stimulus "A" required a response on the left CRTL button while stimulus "B" required a response on the right CRTL button (Go trials). When the target stimulus was presented in a ***bold italic*** font ("***A***" or "***B***"), all reactions had to be inhibited (Nogo trials). When the spatial location of the stimulus matched that of the associated response hand (i.e., letter "A," on the left side of the screen or letter “B” on the rioght side of the screen), the trial was coded as *congruent*, while a mismatch between stimulus location and response hand was coded as *incongruent*. Go trials were presented at a ratio of 7:3 to Nogo trials, while congruent and incongruent trials were featured with the same frequency in both Go and Nogo trials. In total, 720 trials were presented in six equally sized blocks.

### EEG recording and analysis

EEG signals were recorded with 60 Ag/AgCl ring electrodes in an equidistant layout. After data preprocessing with Brain Vision Analyzer (Version 2.2; Brain Products GmbH, Gilching, Germany), the datasets were exported to Matlab (Version R2020b; The MathWorks Inc., MA, United States) for further analyses with the FieldTrip toolbox^64^. The data was segmented in trials of 4 s duration, locked to the onset of the stimulus (− 2000 to 2000 ms), and classified for each trial condition (congruent/incongruent Go/Nogo trials) and intoxication state (sober vs. intoxicated). Only trials with a correct response (Go) or correct omissions (Nogo) were segmented. Automated artifact rejection was applied to remove segments with amplitudes higher than 100 µV or lower than − 100 µV, as well as activity below 0.5 µV over a time of 100 ms. Theta band activity was analyzed by performing time-frequency (TF) decomposition using Morlet wavelets. The average power over the theta frequency band was calculated for each electrode and time point. The segments were then shortened to intervals of 600 ms duration in the pre-trial and within-trial period for further analyses. A duration of 600 ms was chosen because it allows for a minimum of three theta cycles at the central frequency of 5.5 Hz. For the pre-trial interval, the period from − 600 to 0 ms relative to stimulus onset was selected from all trials and conditions. The timeframe from 150 to 750 ms (relative to stimulus onset) was separately extracted for all four trial types in the within-trial period. Cluster-based permutation tests with 1000 Monte Carlo randomizations and a two-tailed cluster-alpha level of *p* = 0.025 were run to identify clusters of electrodes showing significant differences between the intoxicated and sober conditions in both the pre-trial and all four within-trial intervals.

The source reconstruction of theta band activity from the EEG data followed a multi-step beamforming approach used in previous studies^29,53^. First, a Dynamic Imaging of Coherent Sources (DICS)^65^ beamformer was used to identify sources of theta band activity in the brain. In the second step, clusters of theta activity were identified by a Density-Based Spatial Clustering of Applications with Noise (DBSCAN) algorithm^66^, as previously applied^29,67^. Only voxels with relevant source power differences between the intoxicated and sober conditions were selected for subsequent analyses. In the last step, the theta band activity time course in the obtained regions was reconstructed using a Linearly Constraint Minimum Variance (LCMV) beamformer^68^. In the final step, the source-reconstructed theta time course differences in all four within-trial intervals were correlated with the pre-trial theta time course differences. Pearson correlations were subsequently adjusted for the false discovery rate (FDR) using the Benjamini-Hochberg method, resulting in *q *values. Furthermore, we constricted significant correlations to *q* < 0.0001 to use a conservative threshold.

### Ethics approval

The study was approved by the Ethics Commission of the Medical Faculty of the TU Dresden (EK 293082014) and conducted in accordance with the declaration of Helsinki.

## Supplementary Information

Supplementary Information.

## Data Availability

All study data are available upon request to Paul.Wendiggensen@ukdd.de.
