# Supplementary material for: Alcohol-induced deficits in reactive control of response selection and inhibition are counteracted by a seemingly paradox increase in proactive control
Source: Sci Rep. 2023 Jan 19;13:1097. doi: 10.1038/s41598-023-28012-5 (PMC9852446; doi:10.1038/s41598-023-28012-5)
Supplement: Supplementary file 1 — Supplementary Information. [file 41598_2023_28012_MOESM1_ESM.pdf]

Supplemental Material

Title: **Alcohol-induced deficits in reactive control of response selection and inhibition are counteracted by a seemingly paradox increase in proactive control**

Authors: **Ann-Kathrin Stock, Paul Wendiggensen, Filippo Ghin, Christian Beste**

Materials and Methods

Participants

Only males were included in the current study due to the potential risk of undetected pregnancy and confounding effects related to hormonal changes due to the menstrual cycle in females. Subjects were screened for drinking habits (no highly frequent binge drinking, defined as four or more binge drinking events per month). Participants were further required to be within risk level zone I (0-8 points) or zone II (9-15 points) on the AUDIT, as higher scores require counseling or monitoring of drinking habits according to WHO standards [1].

Experimental alcohol administration

A within-subject design was used, where subjects were tested once sober and once intoxicated. The order of appointments was randomized between subjects. N = 19 participants had the intoxicated appointment before the sober appointment, and n = 16 had the sober appointment before the intoxicated appointment. The appointments were at least 48 hours, but no more than seven days apart from each other. Participants were asked to avoid drinking the night before each appointment and to refrain from consuming caffeine, nicotine, guanine, and all other stimulant or sedative substances four hours before the experimental sessions. Participants were additionally asked to stop eating at least three hours before the intoxication session. Further exclusion criteria were the use of illicit drugs (amphetamines, methamphetamine, morphine, and THC), which was tested on both appointments using a "nal von minden Drug-Screen" urine test (nal von minden GmbH, Regensburg, Germany).
The alcohol administration followed a fixed procedure and was based on individual estimates. A version of the Widmark and Watson et al. [2,3] equation was used to estimate the total body water (TBW) in the male body:

$$TBW = 2.447 - (0.09516 \times age) + (0.1074 \times height) + (0.3362 \times weight).$$

Based on the obtained TBW estimate, the individual amount of alcohol was determined as follows:

$$amount\ of\ alcohol\ in\ grams = \frac{TBW \times 1.055 \times max.BrAC}{0.8}$$

The individual amount of vodka (40% alcohol by volume) was calculated to reach a maximal possible breath alcohol concentration (BrAC) of 1.5 ‰ (mg/g). Factoring in a resorption deficit of 20%, participants would reach a mean peak BrAC of 1.2‰ (on an empty stomach). The vodka was diluted with an equal amount of orange juice and served at room temperature. Participants were asked to consume the drink within 30 minutes. During the consumption period and a subsequent waiting period of 30 minutes, the participants watched three episodes of the series *The Big Bang Theory*, which had been chosen because of the lack of extensive discourses on drinking and to help prevent mood swings [4]. According to the manufacturer's instructions, breath alcohol levels were measured immediately before and after the task using the "Alcotest 3000" breathalyzer (Drägerwerk, Lübeck, Germany). This essentially leads to the same results as venous blood analysis using a capillary gas chromatography-headspace technique [5,6]. During

the sober appointment, only water (i.e., no placebo) was provided, as previous piloting had shown that participants could easily distinguish a placebo from alcohol for the planned level of intoxication [7].

Task

Participants were seated in front of a 17" CRT monitor. Stimuli were presented using “Presentation” software (Neurobehavioral Systems Inc., CA, United States). Participants were asked to place their left and right index fingers on the left and right CRTL buttons of a regular computer keyboard, respectively. A white fixation cross and two lateralized white frame boxes (1.1° visual angle from the fixation cross) were displayed on a black background for the entire duration of the experiment. In each trial, a single yellow letter stimulus in "Arial" font (i.e., "A" or "B") was presented either in the left or right white frame box for 200 ms while a distractor stimulus (three white horizontal lines) was shown on the other side. Presentation of the letter "A" required a response on the left CRTL button while stimulus "B" required a response on the right CRTL button. Participants were asked to respond as fast as possible by pressing the corresponding control button when the standard letter target stimuli were presented (Go trials). When the spatial location of the stimulus matched that of the associated response hand (e.g., when the letter "A", requiring a left button press, was presented on the left-hand side of the screen), the trial was coded as *congruent*, while a mismatch between stimulus location and response hand was coded as *incongruent*. Go trials were recorded as hits if the correct response was given in the response time window from 250 to 1000 ms after stimulus onset, while they were recorded as incorrect when the wrong response was given. If no button press was detected within the response time window, the responses were recorded as misses. If no response was given within 500 ms after the Go stimulus onset, a speed-up notice ("Faster!") was displayed above the fixation cross. When the stimulus was presented in a ***bold-italic*** font ("***A***" or "***B***"), all motor reactions had to be inhibited (NoGo trials).

Similarly to Go trials, NoGo trials could be either congruent (“A” on the left or “B” on the right side) or incongruent (“A” on thr right or “B” on the left side). If any response was given within the time interval from 0 to 1200 ms after stimulus onset, it was recorded as incorrect. Go trials were presented at a ratio of 7:3 compared to NoGo trials, while congruent and incongruent trials were featured with the same frequency in both Go and NoGo trials. In total, 720 trials were presented in six equally sized blocks. While the trials were randomly presented, it was ensured that the trial distribution was identical for each block. After each block, participants were able to take a self-timed break. The inter-trial interval was jittered between 1300 and 1700 ms. Overall, the experimental task took approximately 30 minutes to complete.

EEG recording and preprocessing

EEG signals were recorded with 60 Ag/AgCl ring electrodes in an equidistant layout. The ground electrode was located at the coordinates $\theta = 58$, $\varphi = 78$, while the reference electrode was positioned at $\theta = 90$, $\varphi = 90$ (corresponding to electrode position Fpz). Electrode impedance was kept below 5 kΩ. Data were recorded at a sampling rate of 500 Hz. The data sets were pre-processed using Brain Vision Analyzer (Version 2.2; Brain Products GmbH, Gilching, Germany): The data was down-sampled to 256 Hz. The signal was band-pass filtered (IRR) between 0.5 and 40 Hz (with a 48 dB/oct), and a notch filter at 50 Hz was set to suppress line noise. Where necessary, noisy or flat channels were removed before the data were re-referenced to the common average. Pauses and technical artifacts were removed from the data using a manual data inspection. Artifacts like eye blinks, lateral eye movements and cardiovascular activity were removed with an Independent Component Analysis (ICA; Infomax algorithm). A second visual inspection was conducted to ensure the quality of the signal after the back-projection of the removed ICA components.

Beamforming analysis

The source reconstruction of theta band activity from the EEG data followed a multi-step beamforming approach used in previous studies [8,9]. First, a Dynamic Imaging of Coherent Sources (DICS) [10] beamformer was used to identify sources of theta band activity in the brain. After that, a Linearly Constraint Minimum Variance (LCMV) [11] beamformer was used to reconstruct the activity in the previously determined source of activity. Using this approach, the reconstructed time courses of theta band activity in the pre-trial and within-trial intervals can be correlated.

DICS beamforming was applied using common spatial filters derived from the cross-frequency spectrum of a Fast Fourier Transform (FFT) over the averaged theta frequency band (4 – 7 Hz). The common spatial filters were calculated over the two levels of intoxication status (intoxicated and sober). The data were subsequently projected onto an equally spaced 1 cm grid based on the forward model template of the FieldTrip toolbox. Theta power values were extracted for the pre-trial and within-trial intervals. The source power difference between the intoxication and sober condition was calculated by subtracting the latter from the first. The difference was further normalized on the sum of the intoxicated and sober conditions:

$$ratio = \frac{Power_{Intoxicated} - Power_{Sober}}{Power_{Intoxicated} + Power_{Sober}}$$

In the second step, clusters of theta activity in the source power ratio were identified by a Density-Based Spatial Clustering of Applications with Noise (DBSCAN) algorithm [12] as previously applied [8,13]. Only voxels with relevant source power differences between the intoxicated and sober conditions were selected for subsequent analyses following this procedure. Power values were thresholded at the top 1% of the power distribution with labeled regions in the Automatic Anatomical Labeling (AAL) atlas [14], excluding the cerebellum. Epsilon was set to twice the voxel length to ensure detection of neighboring voxels. The identified clusters of high theta power differences were then visually inspected and selected for further analysis based on cluster size (voxels included in the cluster) and anatomical labels.

In the last step, the theta activity time course in the regions obtained by applying the DBSCAN algorithm was reconstructed using an LCMV beamformer [11]. The pre-processed data was multiplied with the spatial filter derived from the covariance matrix of the averaged data in the corresponding trial condition for each cluster. Time-frequency analyses with Morlet wavelets (for parameters, see above) were calculated, and power values were averaged over all voxels in the clusters, resulting in a single theta band activity time course for that cluster. For each cluster, the difference between the intoxicated and sober condition was calculated by subtracting the sober condition theta time course from that of the intoxicated condition. In the final step, the source-reconstructed theta time course differences in each of the four within-trial interval were separately correlated with the pre-trial theta time course differences.

Statistical analyses

Statistical analyses for the behavioral data were performed in IBM SPSS Statistics 27. Mixed-effects ANOVAs were calculated separately for Go trials (error rate and hit response time) and NoGo trials (false alarm rate). Intoxication status (intoxicated vs. sober) and experimental condition (congruent vs. incongruent stimulus/response locations) were used as within-subject factors. Additionally, appointment order (intoxicated first vs. sober first) was used as a between-subject factor for all analyses. The standard error of the mean (SEM) is given as a measure of variability for all descriptive statistics. Where required, p values were Bonferroni-corrected to adjust for multiple comparisons.

References

1. Barbor, T., Higgins-Biddle, J., Saunders, J. & Monteiro, M. *The Alcohol Use Disorders Identification Test Guidelines for Use in Primary Care*. (2001).
2. Widmark, E. M. P. Die theoretischen Grundlagen und die praktische Verwendbarkeit der gerichtlich-medizinischen Alkoholbestimmung /. (1932).
3. Watson, P. E., Watson, I. D. & Batt, R. D. Total body water volumes for adult males and females estimated from simple anthropometric measurements. *Am J Clin Nutr* **33**, 27–39 (1980).
4. Friedman, R. S., McCarthy, D. M., Bartholow, B. D. & Hicks, J. A. Interactive effects of alcohol outcome expectancies and alcohol cues on nonconsumptive behavior. *Experimental and Clinical Psychopharmacology* **15**, 102–114 (2007).
5. Stock, A.-K., Blaszkewicz, M. & Beste, C. Effects of binge drinking on action cascading processes: an EEG study. *Arch Toxicol* **88**, 475–488 (2014).
6. Stock, A.-K. & Beste, C. Binge drinking and the differential influence of ethanol on cognitive control subprocesses: a novel field of neurotoxicology. *Arch Toxicol* **88**, 9–10 (2014).
7. Chmielewski, W. X., Zink, N., Chmielewski, K. Y., Beste, C. & Stock, A.-K. How high-dose alcohol intoxication affects the interplay of automatic and controlled processes. *Addiction Biology* **25**, e12700 (2020).
8. Adelhöfer, N. & Beste, C. Pre-trial theta band activity in the ventromedial prefrontal cortex correlates with inhibition-related theta band activity in the right inferior frontal cortex. *NeuroImage* **219**, 117052 (2020).
9. Dippel, G., Mückschel, M., Ziemssen, T. & Beste, C. Demands on response inhibition processes determine modulations of theta band activity in superior frontal areas and correlations with pupillometry – Implications for the norepinephrine system during inhibitory control. *NeuroImage* **157**, 575–585 (2017).
10. Gross, J. *et al.* Dynamic imaging of coherent sources: Studying neural interactions in the human brain. *Proceedings of the National Academy of Sciences* **98**, 694–699 (2001).
11. Van Veen, B. D., van Drongelen, W., Yuchtman, M. & Suzuki, A. Localization of brain electrical activity via linearly constrained minimum variance spatial filtering. *IEEE Trans Biomed Eng* **44**, 867–880 (1997).
12. Ester, M., Kriegel, H.-P., Sander, J. & Xu, X. A density-based algorithm for discovering clusters in large spatial databases with noise. in *Proceedings of the Second International Conference on Knowledge Discovery and Data Mining* 226–231 (AAAI Press, 1996).
13. Adelhöfer, N., Schreiter, M. L. & Beste, C. Cardiac cycle gated cognitive-emotional control in superior frontal cortices. *NeuroImage* **222**, 117275 (2020).
14. Tzourio-Mazoyer, N. *et al.* Automated anatomical labeling of activations in SPM using a macroscopic anatomical parcellation of the MNI MRI single-subject brain. *Neuroimage* **15**, 273–289 (2002).
